# Leadless Pacemaker Infection Risk in Patients with Cardiac Implantable Electronic Device Infections: A Case Series and Literature Review

**DOI:** 10.3390/jcm12247573

**Published:** 2023-12-08

**Authors:** Lorenzo Bertolino, Letizia Lucia Florio, Fabian Patauner, Raffaella Gallo, Anna Maria Peluso, Antonio Scafuri, Stefano De Vivo, Carmelina Corrado, Rosa Zampino, Giuseppe Ruocco, Antonio D’Onofrio, Emanuele Durante-Mangoni

**Affiliations:** 1 Department of Precision Medicine, University of Campania ‘L. Vanvitelli’, Via de Crecchio, 7, 80138 Napoli, Italy; lorenzo.bertolino@unicampania.it (L.B.); fabianpatauner@gmail.com (F.P.); raffaella.galloo@gmail.com (R.G.); antonio.scafuri97@gmail.com (A.S.); 2 Department of Advanced Medical & Surgical Sciences, University of Campania ‘L. Vanvitelli’, Piazza Luigi Miraglia, 2, 80138 Napoli, Italy; letizialucia.florio@gmail.com (L.L.F.); annapelu11@gmail.com (A.M.P.); rosa.zampino@unicampania.it (R.Z.); 3 Unit of Cardiac Electrophysiology, AORN Ospedali dei Colli-Monaldi Hospital, Piazzale Ettore Ruggieri, 80131 Napoli, Italy; stefano.devivo@tin.it (S.D.V.); corradocarmelina@libero.it (C.C.); donofrioant1@gmail.com (A.D.); 4 Unit of Infectious and Transplant Medicine, AORN Ospedali dei Colli-Monaldi Hospital, Piazzale Ettore Ruggieri, 80131 Napoli, Italy; 5 Unit of Microbiology and Virology, AORN Ospedali dei Colli, Piazzale Ettore Ruggieri, 80131 Naples, Italy; giuseppe.ruocco@ospedalideicolli.it

**Keywords:** leadless pacemaker, CIED infection, reimplantation strategy, infection risk

## Abstract

(1) Background: Leadless pacemakers (LPs) have been proposed as a reimplantation strategy in pacing-dependent patients undergoing cardiac implantable electronic device (CIED) extraction for infection. In this study, we analysed the risk of LP infection when this device is implanted before lead extraction. (2) Methods: This was a retrospective study including patients who underwent LP implantation between 2017 and 2022. Patients were divided in two groups according to whether LP was implanted following CIED extraction for infection (Group 1) or other indications (Group 2). The primary aim was to describe the risk of LP infection. (3) Results: We included in this study 49 patients with a median age of 81 [20–94] years, mostly males (36, 73%). In Group 1 patients, 17 cases (85%) showed systemic CIED infections, and 11 (55%) had positive lead cultures. Most Group 1 cases (n = 14, 70%) underwent one stage of LP implantation and CIED extraction. Mortality rate during follow-up was 20% (nine patients). Patients were followed up for a median of 927 [41–1925], days and no cases of definite or suspected LP infections were identified. (4) Conclusions: The risk of LP infection was extremely low. LP appears as a potential option for reimplantation in this setting and should be considered in pacing-dependent patients at a high risk of CIED infection recurrence.

## 1. Introduction

Cardiac pacing and defibrillation is an established therapy for patients with bradyarrhythmia and heart failure, with about 1 million cardiac implantable electronic devices (CIED) implanted annually worldwide [1]. The most frequent issues with CIED implant are lead-related and require re-intervention in 1% to 5.9% of cases [2,3]. One of the most threatening complications is CIED infection, which results in increased mortality, morbidity, and health care costs [4,5,6,7].

CIED-related infections occur in 1% of patients overall 12 months after the first implantation, with a three-fold increased risk after replacement, upgrade, or revision procedures [8].

To tackle the growing incidence of CIED infections, miniaturized “wireless” cardiac pacing systems, termed leadless pacemakers (LPs), have been designed, where a pulse generator and electrodes are contained in a single totally intracardiac unit, eliminating surgical pockets and long intravascular leads.

Currently, the use of LPs is restricted to selected patients; guidelines and expert opinion recommend LP be used as an alternative to conventional pacemakers (PMs) on a case-by-case basis. In detail, LP should be considered in patients at high risk of infection, such as those with end-stage chronic kidney disease (CKD) on dialysis, or as a reimplantation strategy in patients with previous CIED infection [9,10].

The Micra Transcatheter LP was approved by the European Commission in 2015 and by the FDA the following year. This LP consists of a small single-chamber ventricular pacemaker made of parylene-coated titanium contained in a hermetically sealed capsule with a volume of 0.8 cm^3^. The device is 25.9 mm long, with an outer diameter of 6.7 mm and a total weight of 2.0 g [11].

The device can stimulate only the ventricular chamber; thus, the main indication for its implantation is atrial fibrillation with slow ventricular response. The results of many studies demonstrate that LPs are safe and have a low prevalence of adverse events, including infectious events, compared with conventional PMs [12].

It can be hypothesised that the spread of LP implantation could be associated with higher infection rates than those previously observed. This makes it necessary to conduct a real-life assessment of the impact of LP infections.

Currently, CIED infection represents the main clinical indication for transvenous lead extraction (TLE) [6]. However, the management of extracted patients remains challenging, as the reimplantation procedure must often be adapted case-by-case [11]. Therefore, considering their inherent increased clinical risk profile, patients undergoing TLE can be considered a special population.

The direct implantation of LPs could be a viable alternative for PM dependent patients undergoing TLE for CIED infection, avoiding the problems associated with the use of a temporary device [11]. Emerging literature reports small case series in which the leadless device was implanted during active cardiovascular infection, and most cases have been evaluated during a short follow-up. Current levels of evidence on the outcome of this approach presently appear relatively weak to warrant a strong recommendation in subjects at a high risk of reinfection. Accordingly, it is essential to gather further data on LP infection risks in this clinical setting. Although the technical features of LPs appear to reduce infectious risks, their placement during active infection has to be further analysed by focused studies.

## 2. Materials and Methods

This was an observational, single-centre, retrospective study, in which data were collected on consecutive patients who underwent LP implantation (de novo or after lead extraction) in the Monaldi Hospital between August 2017 and August 2022. All patients gave their informed consent to the anonymous use of their clinical data. Only patients with single-chamber LP (VVI) were included in the study (Micra Transcatheter Pacemaker System (Medtronic, Minneapolis, MN, USA)).

The primary aim was to describe the risk of LP infection at mid-term follow-up by dividing our cohort in two groups. Group 1 included patients in whom an LP was placed (i) as a reimplantation strategy after CIED extraction for infection or (ii) during acute valvular infective endocarditis (IE). Group 2 included patients who underwent LP implantation for indications not related to CIED infection or valvular IE. This group was included in this study for comparison of infection rates in patients without cardiovascular infection. CIED extraction and LP implantation were performed by the same operator in all cases and, in patients with prior CIED, an LP was placed before lead extraction.

A diagnosis of CIED-related IE or CIED infection was made according to EHRA criteria [6], whereas a diagnosis of valvular IE was made according to ESC 2015 criteria [13].

The following data were collected for each case at the time of LP implantation: age, sex, main comorbidities, kidney function (estimated glomerular filtration rate [eGFR], according to CKD-EPI), left ventricle ejection fraction (LVEF), glucocorticoids or other immunosuppressive agent use, previous cardiac valve surgery, history of CIED infection, history of IE, and outcome. The following additional data were retrieved for Group 1 patients: diagnosis of CIED infection or CIED-related IE at admission, causative microorganism (blood and pocket/device/lead culture), timing between CIED extraction and LP implantation, use of temporary pacing, timing between start of antimicrobial therapy, and LP implantation [14].

After hospital discharge, questionnaires were administered to evaluate signs and symptoms of systemic infection requiring medical attention (both as in-patient and out-patient) until the end of the study. In patients with signs and symptoms of systemic infection, data regarding blood cultures and isolated microorganisms, as well as patient outcome, were retrieved. Echocardiography was not performed in asymptomatic patients as a screening test but only in the case of symptoms development.

Descriptive analyses were performed using the Statistical Package for Social Sciences v. 22 (SPSS, Inc., Chicago, IL, USA) statistical software for Windows. Numerical variables are presented as median and interquartile range (IQR), and categorical data are presented as number and percentage.

## 3. Results

We included in this study 49 patients who underwent LP implantation in the study period. The median age was 81 [20–94] years, and most were males (36, 73%). The main clinical features of our patients are displayed in Table 1.

According to baseline conditions, we observed a moderate burden of comorbidities, including systemic arterial hypertension (41, 83%), ischemic heart disease (IHD) (17, 35%), diabetes mellitus (15, 30%), and chronic obstructive pulmonary disease (COPD) (9, 18%). The main indication for LP placement was atrial fibrillation with a slow ventricular response (24 pts, 49%).

During the hospitalization for LP implantation, 20 cases (40%) showed an eGFR below 60 mL/min/1.73 m^2^, with a median creatinine value of 1.2 [0.5–9.1] mg/dL; only 2 patients where on intermittent haemodialysis. Moreover, three patients showed conditions related to immunosuppressive treatment (two for heart transplant and one for psoriatic arthritis). Previous cardiac valve surgery had been performed in 10 patients (20%): five biological prosthetic valves, two mechanical prosthetic valves, one case each of trans-catheter aortic valve implantation (TAVI), Mitraclip procedure, and mitral valve repair.

Regarding Group 1 patients, 17 cases (85%) showed systemic CIED infections (defined as presence of bacteraemia with or without evidence of lead endocarditis). Isolated generator pocket infection (GPI) was present in three cases. Two patients had valvular IE (aortic bioprosthesis and native mitral valve) and one patient had a severe IE simultaneously involving the mitral valve, aortic valve (TAVI), and the CIED. The most common device implanted was dual-chamber PM (12 pts, 60%), followed by CRT-D (3 pts, 15%), single-chamber ICD in one case, and dual-chamber ICD in one case.

Most cases (n = 14, 70%) in Group 1 underwent CIED extraction and LP implantation in one stage, with the LP placed before TLE. In two patients, the LP implantation was performed within 48 h of TLE, whereas in two cases, the LP was implanted >48 h after TLE. Two patients had LP implanted without performing TLE (valve IE without prior CIED). In one case, the procedure was complicated by superior cava vein rupture and massive right haemothorax and haemorrhagic shocks. Therefore, an emergent open-heart surgery with a repair of the superior cava vein was performed, and the patient was discharged alive after 20 days. The median time of antimicrobial therapy before LP implantation was 16 [6.5–30] days.

At the time of LP placement procedures, 11 patients showed increased C-reactive protein (above 0.5 mg/dL), but none was febrile, and all patients had documented clearance of blood cultures. Additionally, 11 (55%) patients had positive lead cultures. Antimicrobial therapy was administered for a median time of 27 [17–32] days after LP placement. The most common administered antimicrobial was daptomycin in 60% of cases. With regard to the reimplantation strategy in patients with CRT-D or ICD, three patients were also implanted with a subcutaneous ICD (S-ICD) simultaneously for secondary prevention of sudden cardiac death, whereas a downgrade to lone PM (LP) was made in the remaining two patients. The characteristics of Group 1 patients are summarized in Table 2.

Patients in Group 2 showed a median age of 84 [20–94] years, and around 80% were male. The most common comorbidities were arterial hypertension (80%), followed by diabetes (28%), COPD (21%), and IHD (17.2%). Only two patients had a history of prior CIED infection. In-hospital mortality in this group was 24%.

During follow-up, none of the 49 patients underwent LP extraction because of infective complications. Overall, eight infections were identified: blood stream infections related to peripheral (two cases) or central (one case) venous catheter, urinary tract infections (two cases, with one patient showing secondary bacteraemia), and lower respiratory tract infections (three cases, all with negative blood cultures). Patients were followed up for a median of 927 [41–1925] days. In detail, the follow-up median time was 403 [34–1666] days for Group 1 patients and 895 [387–1925] days for Group 2 patients.

The mortality rate in our cohort was 20% (nine patients) during the mentioned follow-up. In this subgroup, the median age was 87 [73–96] years, with a median time between LP implantation and death of 927 [394–1015] days. No patient died because of complications related to LP or its implantation; however, we were unable to establish the cause of death in two cases. Deceased patients showed a high rate of comorbidities such as cardiomyopathy, CKD, and diabetes (Supplementary Table S1).

## 4. Discussion

This study on 49 patients with a 2-year median follow-up confirms that the risk of LP infection is very low even when implanted in patients with CIED infections, including immediately before the infected device lead extraction. In our cohort, we did not observe any case of LP infection or cases of systemic infection requiring LP extraction. According to our view, it is extremely important to underline the low infection risk of these devices considering the frail baseline condition of patients who usually undergo LP placement in the presence of several risk factors for subsequent and recurrent infections. Indeed, in our study, most patients presented multiple risk factors for CIED infection, such as immunosuppression (advanced age, corticosteroid therapy), comorbid conditions (CKD, haemodialysis, chronic heart failure, diabetes mellitus), and history of generator replacements.

In the Micra Investigational Device Exemption (IDE) study, 745 patients were enrolled between 2013 and 2015 in 56 participating centres from 19 countries across the globe, and no cases of infection were observed over the 12-month follow-up [15]. In the Micra Post-Approval Registry (PAR) of the Food and Drug Administration, the real-world safety and efficacy of LP was evaluated on 1817 patients, and 3 cases of infection were documented at 30 days follow-up after implantation. In detail, observed were two cases of skin and soft tissue infection at the surgical site in the abdominal wall and one case of sepsis of unclear aetiology. None of these patients required LP extraction, and all were cured with antimicrobial therapy alone. Currently, only two documented cases of LP infection have been reported, to the best of our knowledge [16,17].

The use of LP in patients with prior CIED infection has been evaluated in other studies. For example, El-Chami et al. [18] collected 105 patients (84 PM, 13 ICD/CRT-D) with a history of CIED infection who underwent LP placement in the 30 days after device extraction. A complete extraction was possible in 93.3% of cases, with a mean time between CIED extraction and leadless PM placement of 6 days; 37.1% of patients received an LP during device extraction. In this study, 10 patients died during follow-up. Of those, two patients died because of systemic infections that occurred 14 and 161 days after LP implantation. None of these cases of systemic infection were related to the LP, and the authors state that these devices may be safely used in patients with CIED infection. Moreover, the study demonstrates that the risk of LP infection remains extremely low, including when these devices are implanted during infected CIED extraction. Our findings corroborate the results of the study. However, in our cohort, most patients underwent LP placement before lead extraction. It is important to underline that the risk of infection remains low, including when the LP system is implanted in an infected site. Indeed, lead cultures were positive in more than 50% of our Group 1 patients.

The safety of a reimplantation strategy using an LP is supported by other studies in patients with generator pocket infection and systemic involvement [19,20,21,22,23,24].

Chang et al. collected 17 cases of CIED infection who underwent LP implantation during TLE. Only four patients had positive blood cultures and only two cases showed lead involvement with vegetation demonstrated at trans-oesophageal echocardiography. During follow-up, they did not observe cases of infection relapse (demonstrated by positive blood cultures), and none of those patients underwent antimicrobial therapy for suspicion of LP device infection. Two patients died for reasons unrelated to the LP [19]. This study differs significantly from ours because in our cohort, most patients from Group 1 (17, 85%) had a systemic infection demonstrated by both positive blood cultures and lead vegetation. Therefore, our results suggest that the risk of LP infection remains low, even in this high-risk setting.

Kypta et al. described six cases of LP implantation after CIED extraction for infection. Three patients showed evidence of lead infection. At 6 months of follow-up, the patients did not present any symptoms or signs of systemic infection, and a whole-body PET-CT scan performed 3 months after LP placement did not show an abnormal tracer uptake of the device [20].

Higuchi et al. described 11 cases in which reimplantation after CIED infection was performed with an LP (10 cases of generator pocket infection, 1 case of CIED IE). Two patients had positive blood cultures, whereas in five cases lead culture was positive. Only one patient (with negative blood and lead cultures) underwent LP placement during CIED extraction, whereas the remainder underwent temporary PM placement for a median time of 14 days before LP placement. No cases of device infection were observed at 17 months of follow-up [21].

In the study by Bicong et al. [22], 9 patients underwent LP placement during TLE for CIED infection, whereas the remainder (30 pts) had a mean time between CIED removal and LP implantation of 4 days. Also, in this study with a long follow-up, the authors did not retrieve any cases of LP infection.

Zhang and colleagues [23] presented an interesting case series of eight patients with generator pocket infection in whom LP was implanted immediately after CIED extraction, and no cases of LP infection occurred. However, in this study, data regarding blood and lead cultures were not provided.

In the study carried out in Santa Chiara Hospital, a national referral centre for CIED extraction, the safety and efficacy of LPs after TLE were evaluated. In 18% of patients (15 of 83), the indication for TLE was CIED infection (11 GPI and 4 systemic infections). In this cohort, the timing of LP placement was extremely variable, ranging between 6 days and 208 months (mean reimplantation time of 911 days). As in previous studies, no cases of infection were retrieved in the 504 [28–672] days of follow-up. According to the authors’ opinion, LPs should be considered as a reimplantation strategy in PM dependent patients with local infection and after transoesophageal echocardiography to exclude lead-related IE before TLE [24]. By contrast, our data suggest that LPs can be safely reimplanted in patients with systemic CIED infection even during TLE. The main findings of the studies reviewed are summarized in Table 3.

Consistent data regarding the overall low risk of LP infection also emerge from the study by Garweg and colleagues [25]. They assessed the incidence and outcome of bacteraemia after LP implantation in 155 patients. They observed 15 (9.7%) cases of bacteraemia during follow-up. Risk factors for positive blood cultures were a history of heart valve surgery and CKD requiring renal replacement therapy. The source of infection was detected in nine patients, and four of them showed evidence of mitral and/or aortic valve IE. In these patients, trans-oesophageal echocardiography did not show vegetation on the LP. The device was removed and sent for microbiological examination in two patients who underwent cardiac surgery for valve replacement, and the device culture yielded negative results. In those cases, in which device extraction was not feasible or warranted, a whole-body PET-CT scan was performed without evidence of LP infection.

Overall, the results of our study are in line with the current literature suggesting LPs are much less prone to infection than other intracardiac devices. As underlined before, this feature is extremely important considering that those devices are usually placed in patients at high infection risks. Indeed, in our cohort, we observed a high rate of several risk factors for CIED infection. With regard to the implantation timing, it is important to underline that in our cohort, a large proportion of cases underwent LP placement during CIED extraction for infection. During follow-up, we did not observe any cases of infection and, therefore, we consider LPs a valid reimplantation strategy in this setting. The reasons behind the LP’s low tendency to infection have not been defined yet; however, this feature may be related to the peculiar design. Firstly, an LP is totally intracardiac and, in contrast with traditional PM, does not show a direct communication with skin or subcutaneous tissues that may increase the risk of contamination and infection. Moreover, the LP delivery catheter has a steerable, flexible shaft with a distal end that contains a device cup to hold the device and a recapture cone to retrieve it. Therefore, the device is not directly handled by the operator, thus reducing the risk of possible perioperative contamination [26]. Another important characteristic that we must consider is the size; indeed, an LP has a surface area of 616 mm^2^ that is much less than that of traditional lead. This feature, together with the complete encasement of the device in a collagen-rich matrix that reduces bacterial adhesion and biofilm production, may explain the extremely low risk of infection [27]. In addition, other characteristics that we must consider are the perylene coating, a material having an intrinsic resistance to bacterial adhesion, and the position in the right ventricle where the blood pressure is increased when compared to the right atrium. The limitations of our study include the small sample size and the retrospective design.

## 5. Conclusions

In conclusion, an LP appear to be a safe and efficacious device for reimplantation in pacing-dependent patients with CIED infection who undergo TLE. The intracardiac position, the small surface area, and the complete encasement may partially explain the extremely low risk of infection of these devices.

## Figures and Tables

**Table 1:** Baseline clinical characteristics of the study cohort and the analysed subgroups.

	Total Cohort	Group 1	Group 2
N	49	20	29
Age, years	81 [20–94]	79 [66–87]	84 [20–94]
Male subjects	36 (73)	13 (65)	23 (79)
LVEF ^1^, %	52 [25–70]	49 [30–66]	54 [25–70]
Creatinine, mg/dL	1.2 [0.5–9.1]	1.2 [0.5–9.1]	1.2 [0.6–3]
eGFR ^2^, mL/min/1.73 m^2^	62 [6–132]	54 [6–102]	60 [20–132]
eGFR < 60 mL/min/1.73 m^2^	20 (40)	8 (40)	12 (41)
**Comorbidities:**			
Dialysis	2 (4)	1 (5)	1 (3)
Arterial hypertension	41 (83)	18 (90)	23 (80)
Ischemic Heart Disease	17 (35)	12 (60)	5 (17)
COPD ^3^	9 (18)	3 (15)	6 (21)
Diabetes mellitus	15 (30)	7 (35)	8 (28)
**Type of CIED ^4^:**			
Dual-chamber PM ^5^	14 (28)	12 (60)	2 (7)
Single-chamber ICD ^6^	1 (2)	1 (5)	0 (0)
Dual-chamber ICD	1 (2)	1 (5)	0 (0)
CRT-D ^7^	3 (6)	3 (15)	0 (0)
**Indication for LP ^8^:**			
AF ^9^ with SVR ^10^	24 (49)	8 (40)	16 (55)
SND ^11^	5 (10)	1 (5)	4 (14)
Other	15 (31)	8 (40)	7 (24)
Unknown	5 (10)	3 (15)	2 (7)
Corticosteroid therapy	3 (6)	1 (5)	2 (7)
Previous cardiac surgery	10 (20)	7 (35)	3 (10)
History of CIED infection	10 (20)	8 (40)	2 (9)
Follow-up time, days	927 [41–1925]	403 [34–1666]	895 [387–1925]
Mortality	9 (18)	2 (10)	7 (24)

^1^ LVEF: Left Ventricular Ejection Fraction; ^2^ eGFR: estimated Glomerular Filtration Rate; ^3^ COPD: Chronic Obstructive Pulmonary Disease; ^4^ CIED: Cardiac Implantable Electronic Device; ^5^ PM: pacemaker; ^6^ ICD: Implantable Cardioverter Defibrillator; ^7^ CRT-D: Cardiac Resynchronization Therapy—Defibrillator; ^8^ LP: Leadless Pacemaker; ^9^ AF: Atrial Fibrillation; ^10^ SVR: Slow Ventricular Response; ^11^ SND: Sinus node Dysfunction.

**Table 2:** Characteristics of patients with LP placement during the acute episode of cardiovascular infection (Group 1).

Patient	Age	Sex	History of CIED ^1^ Infection	Reason for Admission	Causative Microorganism	Device Culture	Days of Antimicrobial Therapy before Micra Placement	Timing between CIED Removal and Micra Placement, h	Additional Notes
1	67	M ^2^	Yes	CIED-related IE ^3^	MSSA ^4^	Negative	Unknown	0	CR-BSI ^5^ caused by *P. aeruginosa* after CIED extraction
2	81	F ^6^	No	CIED-related IE	MRSE ^7^	Positive	Unknown	0	
3	86	M	No	CIED-related IE	MRSE	Positive	Unknown	0	
4	87	F	No	CIED-related IE	MSSA	Positive	Unknown	Unknown	
5	86	M	Yes	CIED-related IE	MSSE ^8^	Positive	50	0	
6	78	M	No	CIED-related IE	*P. aeruginosa*	Unknown	30	0	
7	71	M	Yes	CIED-related IE	MSSA	Positive	30	48	
8	83	M	No	CIED-related IE	*E. faecalis*	Positive	22	0	
9	73	M	Yes	CIED-related IE	*E. cloacae*	Positive	17	0	Partial CIED extraction complicated by superior cava vein rupture. UTI ^9^ caused by *E. cloacae*
10	80	F	No	CIED-related IE	*S. hominis*	Positive	15	0	
11	76	F	Yes	CIED-related IE	*C. striatum*/MRSE	Positive	7	0	
12	66	M	No	CIED-related IE	MSSA	Positive	5	0	MSSA-related BSI
13	84	M	No	CIED-related IE	MRSE	Unknown	3	0	
14	68	M	Yes	CIED-related IE	*P. aeruginosa*	Positive	0	0	
15	78	F	No	Valve IE (aortic bio-prosthesis)	*S. gallolyticus*	NA ^10^	15	NA	Complicated UTI by *E. faecium*, BSI by *S. hominis*
16	81	M	Yes	IE on native mitral valve, TAVI ^11^ and CIED	MSSE	Negative	24	48 (with temporary pacing)	
17	73	F	No	Valve IE (native mitral valve)	*S. lugdunensis*	Negative	32	NA	AF ^12^ with low ventricular response requiring Micra implantation 28 days after surgery.
18	82	M	No	GPI ^13^	MSSE	Negative	Unknown	Unknown	
19	81	F	Yes	GPI	Unknown	Negative	Unknown	0	
20	67	M	No	GPI	MSSA	Negative	7	0	

^1^ CIED: Cardiac Implantable Electronic Device; ^2^ M: Male; ^3^ IE: Infective Endocarditis; ^4^ MSSA: Meticillin-susceptible *Staphylococcus aureus*; ^5^ CR-BSI: Catheter-related Blood Stream Infection; ^6^ F: Female; ^7^ MRSE: Meticillin-resistant *Staphyloccucus epidermidis*; ^8^ MSSE: Meticillin-susceptible *Staphyloccucus epidermidis*; ^9^ UTI: Urinary Tract Infection; ^10^ NA: Not Applicable; ^11^ TAVI, Transcatheter Aortic Valve Implantation; ^12^ AF: atrial fibrillation; ^13^ GPI: Generator Pocket Infection.

**Table 3:** Description of the main studies addressing the risk of LP ^1^ infection in patients with CIED-I ^2^ and comparison with our results.

	Total Number	LP Implant during TLE ^3^, n (%)	Mean Time between CIED Removal and LP Implants, Days	Lead Vegetations, n (%)	Positive Blood Culture, n (%)	Positive Lead Cultures, n (%)	Staph. Aureus Infection, n (%)	Follow-Up, Days, Median [IQR]	LP Infections during Follow-Up, n (%)
Chami et al. [18]	105	38 (36)	6	NA ^4^	NA	NA	NA	365 [365–365]	0
Chang et al. [19]	17	17 (100)	0	2 (11)	11 (64)	NA	5 (29)	143 [57–181]	0
Kypta et al. [20]	6	2 (33)	NA	NA	NA	NA	NA	180 [180–180]	0
Higuchi et al. [21]	11	1 (9)	14	1 (9)	2 (18)	5 (45)	4 (36)	510 [510–510]	0
Bicong et al. [22]	39	9 (23) *	4	9 (15)	22 (56)	NA	17 (43)	744 [303–1145]	0
Zhang et al. [23]	8	8 (100) **	0	0	0	NA	NA	NA ^†^	0
Zucchelli et al. [24]	15	0	911	3 (20)	4 (26)	NA	NA	504 [28–672]	0
Present study	20	14 (70)	0	15 (75)	11 (55)	11 (55)	5 (25)	403 [34–1666]	0

^1^ LP: Leadless Pacemaker; ^2^ CIED-I: Cardiovascular Implantable Electronic Device—Infection; ^3^ TLE: Transvenous Lead Extraction; ^4^ NA: Not available. * 1 patient underwent LP placement the day before CIED extraction. ** LP was implanted immediately after lead removal. ^†^ Median follow-up time was not available (shorter follow-up 1 month, longer 10 months).

## Data Availability

Data are made available by the corresponding author upon reasonable request.

## Supplementary Materials

The following supporting information can be downloaded at: https://www.mdpi.com/article/10.3390/jcm12247573/s1, Table S1: Characteristics of patients deceased during follow up after LP implantation.
