# Supplementary material for: Leadless Pacemaker Infection Risk in Patients with Cardiac Implantable Electronic Device Infections: A Case Series and Literature Review
Source: J Clin Med. 2023 Dec 8;12(24):7573. doi: 10.3390/jcm12247573 (PMC10743511; doi:10.3390/jcm12247573)
Supplement: Supplementary file 1 [file jcm-12-07573-s001.zip › jcm-2741530-supplementary.pdf]

Table S1. Characteristics of patients deceased during follow up after LP1 implantation.

	Sex	LP implated during CIED[2] infection	Age	Days between LP implantation and death	Infections during follow-up	Cause of death
**1**	M	No	87	401	No	Acute coronary syndrome
**2**	M	No	91	387	No	Acute aortic syndrome
**3**	M	No	87	1003	No	Stroke
**4**	M	No	83	961	No	Unknown
**5**	F	No	73	1140	No	Unknown
**6**	F	No	96	915	No	Complications of hip fracture
**7**	M	No	93	1027	No	Heart failure
**8**	M	Yes	85	927	No	Lung cancer
**9**	F	Yes	81	162	No	Heart failure

[1]LP: Leadless Pacemaker; [2]CIED: Cardiovascular Implantable Electronic Device
